# Prevalence of fibromyalgia in a low socioeconomic status population

**DOI:** 10.1186/1471-2474-10-64

**Published:** 2009-06-08

**Authors:** Ana Assumpção, Alane B Cavalcante, Cristina E Capela, Juliana F Sauer, Suellen D Chalot, Carlos AB Pereira, Amélia P Marques

**Affiliations:** 1 Experimental Physiopathology Post Graduating Program, School of Medicine, University of São Paulo, São Paulo, Brazil; 2 Department of Physical, Speech, and Occupational Therapy, School of Medicine, University of São Paulo, São Paulo, Brazil; 3 Department of Physical Therapy, Universidade Bandeirantes, São Paulo, Brazil; 4 Statistics Department, Institute of Mathematics and Statistics, University of São Paulo, São Paulo, Brazil

## Abstract

**Background:**

The aim of this study was to estimate the prevalence of fibromyalgia, as well as to assess the major symptoms of this syndrome in an adult, low socioeconomic status population assisted by the primary health care system in a city in Brazil.

**Methods:**

We cross-sectionally sampled individuals assisted by the public primary health care system (n = 768, 35–60 years old). Participants were interviewed by phone and screened about pain. They were then invited to be clinically assessed (304 accepted). Pain was estimated using a Visual Analogue Scale (VAS). Fibromyalgia was assessed using the Fibromyalgia Impact Questionnaire (FIQ), as well as screening for tender points using dolorimetry. Statistical analyses included Bayesian Statistics and the Kruskal-Wallis Anova test (significance level = 5%).

**Results:**

From the phone-interview screening, we divided participants (n = 768) in three groups: No Pain (NP) (n = 185); Regional Pain (RP) (n = 388) and Widespread Pain (WP) (n = 106). Among those participating in the clinical assessments, (304 subjects), the prevalence of fibromyalgia was 4.4% (95% confidence interval [2.6%; 6.3%]). Symptoms of pain (VAS and FIQ), feeling well, job ability, fatigue, morning tiredness, stiffness, anxiety and depression were statically different among the groups. In multivariate analyses we found that individuals with FM and WP had significantly higher impairment than those with RP and NP. FM and WP were similarly disabling. Similarly, RP was no significantly different than NP.

**Conclusion:**

Fibromyalgia is prevalent in the low socioeconomic status population assisted by the public primary health care system. Prevalence was similar to other studies (4.4%) in a more diverse socioeconomic population. Individuals with FM and WP have significant impact in their well being.

## Background

Fibromyalgia (FM) is a frequent rheumatologic disorder worldwide [1-5]. It responds by up of 7% of all primary care consultations, imposing substantial costs to the system [6].

The prevalence of FM ranges from 0.66% to 10.5% [7], with most studies pointing into a prevalence around 2% in the adult population. Prevalence is higher in women (around 3.4%) [8] and in middle-age (5%) [1,4]. Most studies were conducted in developed countries; studies in emerging countries or in under-assisted populations are scarce.

Although the impact of FM in productivity [9,10], disability [11,12] and quality of life [13,14] of sufferers is well documented in the Brazilian population [14,15], only one study assessed its prevalence. This study was conducted in a single city and reported a prevalence of 2.5% [4]. Because Brazil is a continental country, other studies are necessary. A better understanding of the epidemiology of FM is an important step for planning the health care system to properly assist this population, assuring improvements in the quality of care and rational cost-implementation.

The aim of this study was to estimate the prevalence of FM and its symptoms in a middle-aged adult population (35–60 years) registered in the primary health care system of a city in Brazil (Embu City, São Paulo State). The population assisted by this system is of low income. We hypothesized that the prevalence of FM is increased in this low income population, may be because they are exposed to several pain risk factors.

## Methods

### Sample

Our study was conducted in Embu, in the metropolitan area of São Paulo, Brazil. We chose this city because it has similar socio-demographic characteristics of São Paulo city and of other cities in the area. The city has an area of 70 km^2^, with a population of 207,663 inhabitants. Most of its population is of low income as per estimates of Gross Domestic Income (GDI – *per capita *GDI is 3,000 dollars). The Human Development Index (HDI) is 0.772; 7.7% of the adult population is illiterate; overall, mean number of school years is 6.5 [16].

Our sample was non-probabilistic; potential participants were selected from the nine units that form the first access to public primary health care system, from the Brazilian Unified Health System, in the city [16]. For individuals of low-income, the Unified Health System is basically the only option for health care, also granting access to other forms of governmental support. For Embu population, the public health care represents 77% of all health establishments in the city [16].

### Procedures and Methods

For the screening survey, we selected all individuals registered in the nine primary care units during the year of 2003. Potential participants were 35–60 years in the year of 2004. In a pilot study we found difficult to conduct face-to-face interviews with these individuals. Because 73% of the 3,109 registered subjects had telephone at the time of the survey, we conducted the screening interviews by phone.

We attempted to contact all 2,269 potential participants. Around 30% of them could not be contacted because were not available, even though the calls were done more than once for each household, and at different times of the day. The refuse rate was insignificant (>1%). Because our sample size was estimated as being less than 768, we ended this screening phase.

The screening phone interviews were conducted by trained staff (three physical therapists and three students of physical therapy). They assessed the presence of pain (yes/no), time of pain (in months), and pain distribution (which sites of pain). The screening survey intended to triage individuals with pain as it is typically done in studies of this nature.

**Figure 1:**
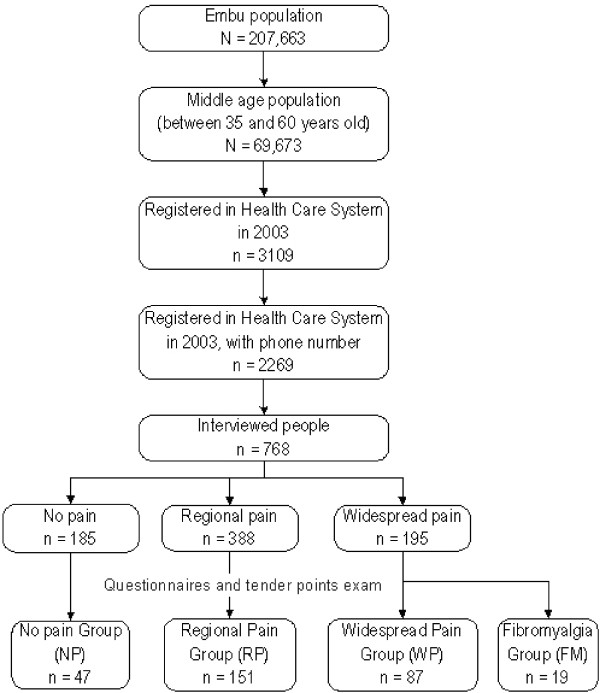
**Flow chart for inclusion procedure and distribution of subjects among groups**.

According to the American College of Rheumatology criteria (ACR), respondents were classified as suffering from widespread pain (WP) when they had pain in the "axial + upper and lower segment + left and right-sided pain" [17]. Those without WP were classified as having regional pain (RP – pain not fulfilling the WP criteria) or no pain (NP). The majority of subjects with pain (92%) had it for more then three months (chronic pain) (Figure 1).

All 768 respondents were invited to participate in a clinical interview, where they would be assessed using standardized questionnaires and exams of potential tender points. Of them, 304 accepted and were seen in one of the participant's primary care units. The volunteers did not receive any financial support.

In the evaluation, we obtained socio-demographic data, as well as pain history. Pain intensity was measured with the Visual Analogue Scale (VAS). Tender points were assessed using the Fischer dolorimeter [18], according to the ACR [17] and Okifuji et al. [19]. Tender points were defined positive if tenderness was present at 2.6 kg/cm^2 ^of pressure [20]. Tests were performed by an experienced physical therapist. Although the ACR criteria recommend manual pressure for determining tender points, the reliability of this method is questionable [21,22], and assessments of pain threshold by dolorimeters have been suggested [21].

Major FM symptoms were assessed by the *Fibromyalgia Impact Questionnaire *(FIQ) [23], which has been validated for Brazilian Portuguese [24]. Because of the high proportion of individuals with low education, the questions were supervised by a researcher.

The screening algorithm for the diagnosis of FM considered the presence and number of tender points and the pain features, as proposed by the ACR (Figure 1).

This study was reviewed and approved by the local Ethics Committee and informed consent was obtained.

### Data Analysis

The data were coded using Excel for Windows (version 2002) and Analyze-it (version 2000), MINITAB 14, SAS System V8 and Statistica V7.

The prevalence of FM was estimated based on Bayesian Analysis. By using this method, phone interviews could be used to estimate the prevalence of widespread chronic pain. Furthermore, this analysis better address several of the biases in clinical research, allowing the use of a bigger sample (n = 768) in many of the estimates [25].

Data obtained from the FIQ were tested for normality using the Shapiro-Wilk test. Groups were compared using the Kruskal-Wallis Anova and multiple variance analysis. The significance level was 0.05.

## Results

Most of the 768 subjects participating in the screening phone interview were women (77%), married and of low educational level. Table 1 presents demographic data for participants in the phone and clinical assessments.

**Table 1:** Demographic data of participants in the phone interview (n = 788) and on the in-person assessments (n = 304).

**Phone Interview**	**No Pain**	**Regional pain**	**Widespread pain**	
	**(n = 185)**	**(n = 388)**	**(n = 195)**	
**Gender**				
Female	123 (66%)	294 (76%)	173 (88%)	
Male	62 (34%)	94 (24%)	22 (12%)	
**Age (years)**				
Female	57.5 (6.8)	48.8 (7.1))	48.8 (6.7)	
Male	47.8 (8.1)	49.5 (7.5)	51.5 (7.2)	

**Clinical Assessment**	**No Pain**	**Regional pain**	**Widespread pain**	**Fibromyalgia**
	**(n = 47)**	**(n = 151)**	**(n = 87)**	**(n = 19)**

**Gender**				
Female	30 (64%)	117 (77%)	78 (90%)	19 (100%)
Male	17 (36%)	34 (23%)	9 (10%)	0 (0%)
**Age (years) (Mean/SD)**				
Female	47.4 (5.6)	49.6 (6.9)	49.1 (6.8)	50.8 (6.5)
Male	50.8 (7.4)	51.6 (6.0)	51.4 (7.3)	-
BMI* (kg/m^2^) (Mean/SD)				
Female	28.1 (5.9)	27.8 (6.1)	27.4 (4.9)	27.4 (4.5)
Male	27.5 (4.4)	27.0 (3.5)	27.1 (2.5)	-
Total	27.9 (5.3)	27.6 (5.5)	27.3 (6.8)	27.4 (4.5)
**Occupation**				
Retired	2 (4.3%)	24 (15.9%)	12 (13.7%)	4 (21.1%)
Unemployed	2 (2.3%)	6 (4.0%)	4 (8.5%)	1 (5.3%)
Household work	24 (44.2%)	66 (43.7%)	44 (56.4%)	10 (52.7%)
People who work mainly standing up**	17 (35.1%)	41 (27.2%)	21 (24.1%)	4 (21.1%)
People who work mainly sitting down***	6 (12.8%)	12 (7.9%)	6 (6.9%)	0 (0.0%)

** For example: cooker, bodyguard, carpenter etc. *** For example: driver (bus, taxi), secretary etc.

Using the Bayesian approach, the prevalence of widespread chronic pain was 24%, with a 95% credibility interval (CI) [21%; 27%]. Among individuals with WP the prevalence of FM was 18% (95% CI = 11% – 25%). For this calculation, the Beta distribution parameters were 0.1 to 0.4 (*a priori*) and 19.1 to 87.4 (*a posteriori*). The beta distribution assumes continuous.. probability distributions defined on the interval [0, 1]. The *a priori *parameters was defined based on known proportions of WP and FM and the *a posteriori *parameters was calculated based on the number of FM and WP subjects. Accordingly, the prevalence of FM was defined as a function of WP probability and proportion of individuals with WP who also endorsed FM. It was 4.4% (95% CI = 2.7%–6.3%).

### Fibromyalgia symptoms

The items of the FIQ questionnaire were significantly different among the groups, except for physical function and work missed. Individuals with FM were the most impaired. However, in multiple variance analysis, FM and WP were similar, and significantly different than RP and NP.

Individuals with NP had significantly less impairment, as compared to the other groups, in several domains including working for job ability, pain, fatigue, morning tiredness, stiffness and depression. Individuals with RP were less impaired than WP in the domains of well, fatigue, morning tiredness and anxiety, and different from FM for fatigue and stiffness. Individuals with WP and FM had no significant differences in the assessed domains in multiple variances analysis. Pain, as assessed by the VAS, was significantly higher in the FM group, followed by in those with WP (p < 0,001) (Table 2).

**Table 2:** Fibromyalgia symptoms assessed by the Fibromyalgia Impact Questionnaire (FIQ) and by the Visual Analogue Scale (VAS).

**SYMPTOMS**	**No Pain**	**Regional Pain**	**Widespread Pain**	**Fibromyalgia**	
	**(n = 47)**	**(n = 151)**	**(n = 87)**	**(n = 19)**	**P value**
		
	*Mean (SD)*	*Mean (SD)*	*Mean (SD)*	*Mean (SD)*	
**Pain VAS**	2.2 (3.0)	5.1 (3.2)	6.1 (2.9)	7.9 (1.8)	<0.001*

**FIQ**					
*Physical Function*	9.3 (5.9)	11.2 (5.8)	10.8 (5.4)	12.4 (7.7)	0.12
*Feeling Well*	4.8 (2.7)^a, c^	3.7 (2.8)^b^	2.5 (2.5)^b, c^	2.0 (2.6)^a^	<0.001*
*Work Missed*	0.0 (0.1)	0.2 (0.9)	0.1 (0.6)	0.0 (0.0)	0.26
*Job Ability (VAS)*	2.4 (2.8)^a^	5.1 (3.1)^b^	5.7 (3.0)^b^	6.6 (2.8)^a^	<0.001*
*Pain (VAS)*	2.2 (2.8)^a^	5.7 (3.0)^b^	6.3 (2.5)^b^	7.7 (1.8)^a^	<0.001*
*Fatigue (VAS)*	3.1 (3.1)^a, c, d^	5.7 (2.9)^b, d^	6.9 (2.5)^b, c^	7.5 (2.3)^a^	<0.001*
*Morning Tiredness (VAS)*	2.3 (2.3)^b, d, e^	4.1 (3.1)^a, c, e^	5.8 (2.8)^c, d^	6.6 (3.0)^a, b^	<0.001*
*Stiffness (VAS)*	1.8 (2.3)^b, c, d^	5.3 (7.9)^a, d^	5.9 (3.0)^c^	7.5 (2.5)^a, b^	<0.001*
*Anxiety (VAS)*	4.3 (3.3)^a, c, d^	6.3 (2.9)^b, d^	7.6 (2.3)^b, c^	7.6 (2.5)^a^	<0.001*
*Depression (VAS)*	3.4 (3.1)^a, b, c^	5.4 (3.2)^c^	6.2 (2.8)^b^	7.0 (2.5)^a^	<0.001*

* statistically different for α = 0,05, Kruskal Wallis Anova

^a, b, c, d, e ^in pairs identify which groups are statistically different between, Multiple Comparison Test

## Discussion

In our study, the prevalence of FM in a middle-aged adult population of a low socioeconomic population was 4.4% and all cases were women. The prevalence was higher than what has been reported elsewhere. In most studies the prevalence ranges from 2% to 4% [8,13], but important discrepancies exist [26-28].

The high prevalence of FM found in our study may be explained by the age of our inclusion sample (35 – 60 years old), when fibromyalgia is more frequent, as shown in studies that enrolled other age categories, such as 18 or 20 and 70 or 80 years old [1,4,8,13]. Indeed, studies consistently show that FM is more common in middle-aged individuals (Table 3) and is less prevalence in the youngest and the elderly. Carmona et al. 2001 [1] found prevalence of FM being 1.6% from 30–39 years, increasing to 4.9% in the forth decade and then decreasing to 3.7% between 50 and 59 years old. Senna et al., in a study conducted in Brazil found the prevalence of 2.5% [4] However, in those of 35–54 years old, the prevalence was 5.5% [4.2%; 6.8%], numerically higher than ours (4.4%, 95% CI = 2.6%–6.3%).

**Table 3:** Fibromyalgia prevalence in literature in populations from 30 and 59 years.

**Authors**	**gender**	**Prevalence for age classes (years)**					
		
		30–39	35–44	35–54	45–54	40–49	50–59
Carmona et al.[1]	Both	1.6%	-	-	-	4.6%	5.7%
Senna et al.[4]	Both	-	-	5.5%	-	-	-
Topbas et al.[29]	Female	3.5%	-	-	-	5.0%	10.1%
Wolfe et al.[8]	Female	2.0%	-	-	-	3.4%	5.6%
White et al.[13]	Female	-	5.5%	-	6.6%	-	-

Although the prevalence of FM in our casuistic was very similar to what had been found in other studies, we found a higher prevalence of widespread chronic pain (24%, 95% CI = 21–27%). Croft et al. [30] reported prevalence of 11.2%, later confirmed by Storozhenko et al. [31]. This discrepancy may be due to the fact we enrolled a high-risk population for chronic pain. Both middle age [32] and female gender are known risk factors for chronic pain [33-35], determined by biological [36] and social factors [32,37,38]. Increased weight is also a risk factor for pain, especially in women [32]. Furthermore, low-income individuals are more likely to work in manual functions, which may also facilitate injuries and pain. Wijnhoven et al. [39] related that non-paid work (e.g. household work) is per se a risk factor for pain [33,36]. Other studies report an inverse association between education and chronic pain [33,36]. Accordingly, our sample should be considered of high risk for widespread chronic pain (as shown in Table 1).

The last assumption is supported by the fact that several studies showed an inverse relation between presence of musculoskeletal symptoms and socioeconomic status [40]. However, of interest is that these factors did not determine a higher prevalence of FM in our study. Another factor explaining the high prevalence of widespread chronic pain was the screening interview being conducted over the telephone, forcing reliance on self-report.

We also assessed the main symptoms of FM in the population. Although not considered in the ACR criteria, they define the essence of the syndrome [41,42] and add to its disability [13] and negative impact on quality of life [14]. We used the FIQ to assess the impact of FM symptoms in individuals not fulfilling de ACR criteria. Some symptoms were common in the other groups as well. As compared to individuals with NP, there were significant differences for all FIQ items, except for "physical function" and "work missed. However, the multiple variances analysis showed that, for the majority of FIQ items, NP and RP were similar and statistically different than the groups WP and FM (which, in turn, where not different).

Accordingly, we notice that although WP and FM have different criteria and definitions, the intensity of the symptoms is similar. It may be that individuals with WP, due to the physical and emotional aspects of pain [43], decrease physical and social activities and become more sedentary [44]. Eventually this may predispose to anxiety, depression and tiredness which, according to Croft et al. [30], may then predispose to FM.

Alternative, it may be that many individuals with WP have unclassified FM. As with FM, individuals with WP have chronic pain in different regions of the body. However, they have less than 11 tender points. It has been suggested that experienced rheumatologists often diagnose WP as FM, even in the absence of tender points [45]. Indeed, recent discussions question whether the ACR Criteria adequately capture the essence of FM. Symptoms perhaps could be included in the diagnosis again [41,42] as had been used by Smythe & Modollfsky [46].

Being so prevalent in the population assisted by the primary care public system, WP and FM are certainly of economic importance, suggesting that the health professionals should be careful in assessing these disorders. Future studies should be developed to assess the adequacy of the ACR criteria in the primary care.

Our study has limitations. Our sample was formed among people registered in the Public Health Care System of Embu, and the data may not be extrapolated. Several biases may exist in the screening interview including lack of telephone or inability to be contacted. The screening survey was established based on methodological and economical feasibility. Some of these biases were at least partially addressed by the Bayesian Analyses. Nonetheless, we suggest that future studies addressing the low-income population account for inherent problems of dealing with this stratum, including education limitations (difficulties in understanding certain questions or demands, need for cross-cultural adaptation of the instruments, etc). Finally, it may be that the personal assessments should be done in the household of the participants, to mitigate economic barriers (e.g. lack of money for transportation, need to work on weekends). Nonetheless, our findings should not be discrepant to what happens in similar cities.

## Conclusion

Our findings point for the importance of FM to the public health system, and highlight the need of preparing public policies to prepare the system to the demand posed by FM, and also to provide adequate care aiming to improve the daily life activities and the quality of life, as recently suggested by Blyth [47]. These policies are especially important for people of low income, who depend on the government health care system. Because classification of FM was done using the ACR Criteria proposed in 1990, future studies are necessary to discuss the better criteria to account for the syndrome complexity.

## Competing interests

The authors declare that they have no competing interests.

## Authors' contributions

AA took part of acquisition and interpretation of data and of manuscript writing. ALC, SDC and CEC made substantial contributions for acquisition and interpretation of data. JFS participated in the acquisition and interpretation of data, in drafting the manuscript and in revising it critically. CAB conducted the Baeysian analyses and made essential contributions for interpretation of data. APM is responsible for the conception and design of the study, has participated in acquisition of data and revised the manuscript critically. All authors read and approved the final manuscript.

## Pre-publication history

The pre-publication history for this paper can be accessed here:


